# Machine learning reduced workload with minimal risk of missing studies: development and evaluation of a randomized controlled trial classifier for Cochrane Reviews

**DOI:** 10.1016/j.jclinepi.2020.11.003

**Published:** 2021-05

**Authors:** James Thomas, Steve McDonald, Anna Noel-Storr, Ian Shemilt, Julian Elliott, Chris Mavergames, Iain J. Marshall

**Affiliations:** a EPPI-Centre, UCL Social Research Institute, University College London, London, UK; b Cochrane Australia, School of Public Health and Preventive Medicine, Monash University, Melbourne, Australia; c Radcliffe Department of Medicine, University of Oxford, Oxford, UK; d Cochrane, London, UK; e Department of Infectious Diseases, Monash University and Alfred Hospital, Melbourne, Australia; f School of Population Health & Environmental Sciences, Kings College London, London, UK

**Keywords:** Machine learning, Study classifiers, Searching, Information retrieval, Methods/methodology, Randomized controlled trials, Systematic reviews, Automation, Crowdsourcing, Cochrane Library

## Abstract

**Objectives:**

This study developed, calibrated, and evaluated a machine learning classifier designed to reduce study identification workload in Cochrane for producing systematic reviews.

**Methods:**

A machine learning classifier for retrieving randomized controlled trials (RCTs) was developed (the “Cochrane RCT Classifier”), with the algorithm trained using a data set of title–abstract records from Embase, manually labeled by the Cochrane Crowd. The classifier was then calibrated using a further data set of similar records manually labeled by the Clinical Hedges team, aiming for 99% recall. Finally, the recall of the calibrated classifier was evaluated using records of RCTs included in Cochrane Reviews that had abstracts of sufficient length to allow machine classification.

**Results:**

The Cochrane RCT Classifier was trained using 280,620 records (20,454 of which reported RCTs). A classification threshold was set using 49,025 calibration records (1,587 of which reported RCTs), and our bootstrap validation found the classifier had recall of 0.99 (95% confidence interval 0.98–0.99) and precision of 0.08 (95% confidence interval 0.06–0.12) in this data set. The final, calibrated RCT classifier correctly retrieved 43,783 (99.5%) of 44,007 RCTs included in Cochrane Reviews but missed 224 (0.5%). Older records were more likely to be missed than those more recently published.

**Conclusions:**

The Cochrane RCT Classifier can reduce manual study identification workload for Cochrane Reviews, with a very low and acceptable risk of missing eligible RCTs. This classifier now forms part of the Evidence Pipeline, an integrated workflow deployed within Cochrane to help improve the efficiency of the study identification processes that support systematic review production.

## Highlights

• Systematic review processes need to become more efficient.
• Machine learning is sufficiently mature for real-world use.
• A machine learning classifier was built using data from Cochrane Crowd.
• It was calibrated to achieve very high recall.
• It is now live and in use in Cochrane review production systems.

**Key findings**

• Manual workload can be saved by using a machine learning classifier
• The risk of missing eligible studies is accepta bly low.

**What this adds to what was known?**
• Machine learning tools are sufficiently mature to be used in real-world scenarios.
• It is possible to build a machine learning classifier to identify RCTs that is sufficiently reliable to be deployed in live workflows.

**What is the implication and what should change now?**
• Where possible, systematic reviewers should use this classifier to make their work more efficient.

## 1 Background

Cochrane is a leading producer of systematic reviews, with more than 8,000 currently published in the Cochrane Library [1]. These reviews incorporate the results of tens of thousands of randomized controlled trials (RCTs) and other primary studies. The manual effort invested in identifying primary studies eligible for inclusion in these and other systematic reviews is vast. Author teams and information specialists typically search a large number of bibliographic databases to find the comparatively small number of studies eligible to be included [2]. These searches are sensitive to identify as many relevant studies as possible but therefore yield large number of irrelevant records, which are then screened manually by author teams. This is a time-consuming and therefore costly process, especially when all records are checked by at least two people to aid reliability. With the rapidly increasing volume of research being conducted and published [3], systematic reviews tend to be resource-intensive projects, which can take years to complete. As a consequence, many important research questions are not covered by systematic reviews, and it is increasingly difficult to maintain an up-to-date synthesized evidence base [4]. This is a waste of global investment in research, leading to suboptimal decision-making and poorer health outcomes [5].

Automation has been proposed as one possible solution to reduce the manual burden of many systematic review tasks [6]. For example, machine learning classification algorithms (“classifiers”) can “learn” the eligibility criteria of a review through exposure to a manually classified set of documents, thus reducing the human effort required to find relevant studies [7].

To date, most automation approaches operate at the level of individual reviews [8,9], rather than addressing structural deficiencies in research curation [10]. This paper describes an important component in an alternative approach, which aims to improve the efficiency of study identification across multiple systematic reviews of RCTs. The system comprises (1) database searching, (2) machine learning, and (3) crowdsourcing (via the Cochrane Crowd citizen science project) to populate an existing database of RCTs (CENTRAL) [11]. The interlinked system or “workflow” is known as the Cochrane “Evidence Pipeline.” Here we describe the machine learning component of the Pipeline workflow; the other components (the Cochrane Crowd and a Centralised Search Service) are detailed elsewhere [12,13]. The reason that this is so beneficial for Cochrane Reviews is twofold. First, on the basis that RCT study designs can be ethically implemented to produce results capable of supporting causal claims about the beneficial effects of the large majority of health care interventions evaluated in Cochrane Reviews, approximately 90% of Cochrane Reviews aim to include only RCTs. Thus, the capability to efficiently identify studies with designs at scale will generate large corollary cost savings and efficiency gains in review production and updating systems across thousands of Cochrane Reviews, reducing research waste. Second, searches conducted for Cochrane and non-Cochrane health and nonhealth systematic reviews of RCT evidence also retrieve many records of studies that are not RCTs (often well over 50%). Thus, the capability to automatically exclude non-RCTs from manual checking in such reviews will reduce manual workload (because, even if they are about the right topic, the fact that they are not RCTs means that they are ineligible for inclusion), with corollary cost savings and efficiency gains.

We have previously described methods for automatically identifying RCTs from research databases [8]. In that evaluation, we found machine learning classification systems are more accurate than manually crafted Boolean string searches of databases (the current standard practice). Yet, showing higher accuracy in a validation study is not sufficient to ensure new technologies are adopted in practice. We have engaged with the Cochrane Information Retrieval Methods Group (IRMG) with whom we agreed additional requirements for this technology to be adopted by Cochrane. First, the classifier must recall at least 99% of RCTs (a more stringent threshold than we had applied in our previous work) [8]. Second, the classifier should provide an indicative probability score to users. Third, an additional assessment should be done on whether the classifier would be at risk of missing any of the studies included in existing Cochrane Reviews. In this article, we describe the development, calibration, and evaluation of a machine learning classifier designed to meet these requirements, which has subsequently been adopted by and deployed within Cochrane.

## 2 Materials and methods

### 2.1 Cochrane Evidence Pipeline workflow

Cochrane publishes a database of RCTs that are relevant for current or potential future reviews (CENTRAL), with an administrative interface for Cochrane users, known as the Cochrane Register of Studies [11]. Although a rapidly increasing minority of reviews synthesize nonrandomized research designs (including qualitative and quasiexperimental studies), CENTRAL focuses on RCTs, which currently remain the basis of the large majority of published Cochrane Reviews. We likewise focus our efforts on the discovery of RCTs.

We seek to benefit from efficiencies in two ways. First, current practice is to identify RCTs through searches of bibliographic databases using highly sensitive RCT filters. Such filters have *low precision*, retrieving as many as 20 non-RCTs for every true RCT [12]. These irrelevant articles then need to be manually screened and removed. Second, the same studies are retrieved and assessed multiple times by different people across the global systematic review workforce. The Pipeline therefore aims to avoid this duplication of effort by facilitating the reuse of previous assessments as to whether a given report describes, or does not describe, an RCT.

**Fig. 1:**
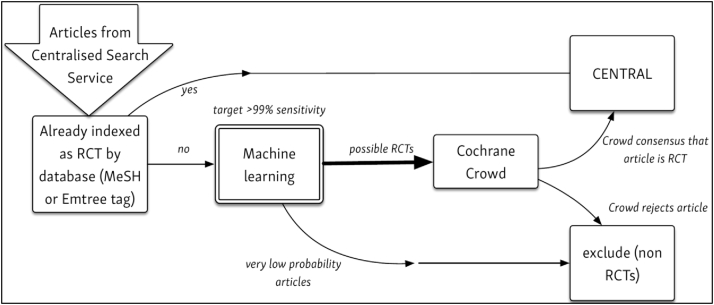
The Cochrane Evidence Pipeline workflow, depicting the flow of records from the centralized search service, through machine and crowd classification services to the CENTRAL database.

Fig. 1 depicts the role of machine learning within the Pipeline. To populate CENTRAL, Cochrane regularly searches a range of online resources (e.g., biomedical literature databases) through the “Centralised Search Service,” which is described elsewhere [12]. Abstracts of these candidate articles (of which the majority are not RCTs) are “fed” into the top of the Pipeline. (The scope and detail of these searches is described here: https://www.cochranelibrary.com/central/central-creation). The machine learning classifier (described in this article) is used to filter out records that are highly unlikely to be an RCT study report. The remaining articles are then handed over to the Cochrane Crowd, which filters out all further records that do not report an RCT [13]. Finally, the remaining articles (which should all describe RCTs) are stored in CENTRAL. Crowd “labels” are also used to update the machine learning algorithm so that it becomes more accurate at distinguishing between relevant and irrelevant records (see Section 2.7).

### 2.2 Data sets and their role in this study

High-quality data sets are vital for the development, calibration, and evaluation of reliable machine learning classifiers. Most evaluations of such classifiers use a single data set, which is split at random between “training” and “test” data (e.g., with 70% of the data reserved for training). The training data are used to estimate the model parameters, and the test data are used to evaluate its performance. However, although a single data set evaluation can provide estimates of classifier performance that have strong internal validity, it cannot tell us how well a classifier will perform in the real world, where data may come from sources that differ in important ways from those used to produce this data set. As outlined by Nevin, it is important to consider the external validity of machine learning models before deployment [14]. Here, we examined external validity in terms of whether the use of our machine learning model would risk missing RCTs included in Cochrane Reviews.

We therefore used three distinct data sets in the present study:

• *Training* data, from which the machine learning models are built;
• *Calibration* data, on which the threshold for determining the cutoff between “RCT” and “non-RCT” classifications was based; and
• *Validation* data, on which the calibrated classifier was evaluated.

**Fig. 2:**
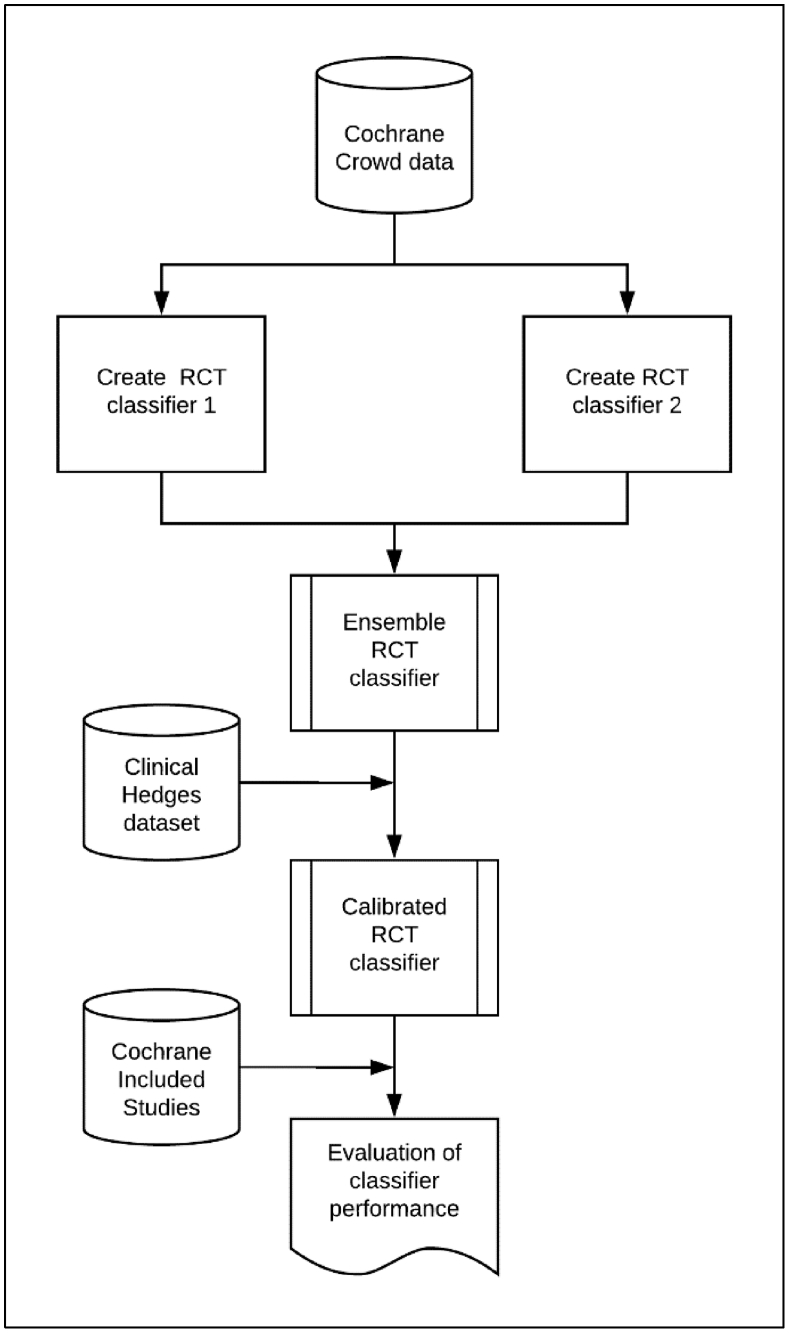
Development and evaluation of the classifier, showing where the various data sets were used in the classifier development process.

Fig. 2 summarizes the contribution made by each data set that we now describe in detail.

### 2.3 Training data

The data set used to train the classifier comprises a corpus of 280,620 title–abstract records retrieved from Embase using a highly sensitive search for RCT (https://www.cochranelibrary.com/central/central-creation). This search has been carried out each month since January 2014 for the purpose of identifying relevant studies for inclusion in CENTRAL (see Section 2). In this study, we used records retrieved between January 2014 and July 2016 inclusive. During this period, any records indexed with the Emtree headings “Randomized controlled trial” or “Controlled clinical study” were automatically marked for inclusion in CENTRAL, without any manual checking, on the basis that this rule produced a false positive rate for identifying reports of RCTs that was judged sufficiently low. To account for the historical use of this rule, records with these specific Emtree headings were also excluded from our training data set. Because obvious RCTs and obvious non-RCTs had already been filtered out of this data set (using Emtree headings and the sensitive search filter for RCTs, respectively) before we used it to train the classifier, the data set therefore comprises records that are, on average, more difficult to classify according to whether or not they report an RCT, compared with an unfiltered sample from the raw database.

Next, each record in the training data set was labeled by Cochrane Crowd members according to whether it reported an RCT (*n* = 20,454) or not (*n* = 260,166). Each record was labeled by multiple Crowd members, with the final Crowd decision being determined by an agreement algorithm; Noel-Storr et al. report that the Crowd recall and precision for identifying RCTs both exceeded 99% [13].

This data set (“Cochrane Crowd data” in Fig. 2) has characteristics that make it highly suitable for training a machine learning classifier: it is both large—so represents a wide range of instances of both the positive and negative classes (i.e., RCTs and non-RCTs)—and also very accurately labeled. However, it also comprised records added to Embase during a relatively short (<3 years) period, which could limit the generalizability of the resulting machine learning classifier.

### 2.4 Calibration data

When machine learning classifiers are used for prediction, they output a score (often scaled to be bounded by 0 and 1) that gets assigned to each title–abstract record, with a higher value representing an increased likelihood that the record reports an RCT. However, to use the classifier to reduce manual screening workload, we also needed to set a threshold score below which records (unlikely to be RCTs) are discarded and conversely above which records (possible RCTs) are retained for manual screening. Higher score thresholds can be expected to lead to a higher prevalence of reports of RCTs (true positives) among fewer retained records (i.e., higher precision) but at the expense of having discarded some reports of RCTs (false negatives) with scores below the threshold (i.e., lower recall). Conversely, a lower threshold can be expected to lead to lower precision but higher recall.

We sought the advice from the Cochrane IRMG and were advised that the classifier would need to have a threshold score calibrated to retrieve at least 99% of relevant RCT study reports to be adopted for use in Cochrane and also that achieving this high level of recall should be prioritized over any reductions in manual screening workload. These specifications reflect the strong aversion that we have, when conducting systematic reviews, to inadvertently failing to identify studies that should be included.

The Clinical Hedges data set (Fig. 2) was built during 2000 and 2001 for the purposes of testing and validating sensitive search filters for RCTs [15]. It contains 49,028 title–abstract records manually identified and selected by information specialists using a combination of hand search and electronic search methods. Corresponding full-text reports of all records were manually checked to ascertain with confidence whether or not each reported an RCT, making this a highly accurate data set for our current purpose. Three records from this data set were no longer available in PubMed, so our final calibration data set comprised 49,025 PubMed title–abstract records, of which 1,587 reported an RCT (and the remaining 47,438 did not report an RCT).

It was more demanding to calibrate our RCT classifier on this data set (compared with using a proportion of records held back from the Cochrane Crowd data set) because (1) the records are older and are less likely to have a consistent reporting structure for RCTs because the study reports were published only a few years after the CONSORT statement (and before the latter became widely used) [16] and (2) the Clinical Hedges Team's assessments were based on full-text reports, and there is no indication in some of the corresponding titles and abstracts that they actually report an RCT. We used this data set to identify the threshold for achieving 99% recall and thereby calibrate our classifier, and we also present results concerning the precision with which these records can be identified.

### 2.5 Validation data

As described previously, this machine learning classifier was primarily designed to identify records of study reports potentially eligible for inclusion in Cochrane Reviews. We therefore validated the classifier using a third data set to determine whether the desired level of 99% recall (calibrated using the Clinical Hedges dataset) could be achieved in practice. This validation data set (“Cochrane Included Studies” in Fig. 2) comprises title and abstract records of all study reports included in Cochrane Reviews in which eligible study designs are restricted to “RCTs only,” published up to April 2017. The data set comprises 94,305 records of 58,283 included studies across 4,296 Cochrane Reviews. Although it could be assumed that the vast majority of these records report an RCT, in practice, we found that some records of included study reports did not report an RCT (e.g., they reported a meta-analysis of RCTs, a related editorial, or personal correspondence). These records were retained in the validation set, as removing them all would have required the manual screening of all records.

### 2.6 Excluded data

Articles without an abstract (i.e., title-only records) may contain insufficient information for accurate machine (or human) classification. However, title-only records that include the words “randomised controlled trial” (as per CONSORT guidance) should be labeled correctly by a classifier. In consultation with the IRMG and in the light of manual assessment of records with some content in their abstract field (but not a full abstract), we determined that pragmatic cutoffs for including a record in the training, calibration, or validation data sets would be set at 400 characters as a minimum abstract length and 15 characters as a minimum title length. These cutoffs aimed to balance the need for sufficient text to be present in the abstract field for the machine learning to operate while not referring too many records for manual assessment. It is important to note that, in the Cochrane Evidence Pipeline workflow (mentioned previously), all records with title and/or abstract fields that have fewer characters than the minimum cutoff are referred for manual screening by members of the Cochrane Crowd. When the minimum character cutoff is applied to the Cochrane Included Studies data set, the final number of studies in the evaluation falls to 44,007.

### 2.7 Machine learning methods for RCT identification

Machine learning describes a group of algorithms that seek to “learn” to do a task by exposure to (typically large amounts of) data. The approach we used here can be described as *supervised machine learning:* meaning that the algorithm is “trained” on articles for which the true label is already known. Although the current state-of-the-art approach for text classification is the use of neural network models, we have previously found that support vector machine (SVM) models (and specifically *ensembles* [ensembling describes a strategy of using multiple machine learning models together, with the aim of improving performance compared with any model individually] of multiple of SVM models) were similarly accurate for high-sensitivity document recall [8]. SVMs are less computationally intensive than neural models and therefore can run quickly and without the need for any special computer hardware. The final Cochrane RCT Classifier model also needed to be deployed in a live Web service that might need to cope with heavy user demand. For these reasons, we chose SVMs for the present study. We refer the interested reader to a detailed description of machine learning methods as applied to abstract classification [8]. In our previous work, we incorporated metadata from the database describing study design into our models (the Publication Type tag in MEDLINE, which is manually added by MEDLINE staff, often several months after publication). However, as the Evidence Pipeline retrieves mainly very new records, which usually lack this metadata, we used a model that uses titles and abstract text without additional metadata.

We used the *bag-of-words* approach, in which each title–abstract record is represented as a vector of 0s and 1s, depending on the presence or absence of each unique word from the article set vocabulary [8]. These vector representations are then used to “train” (i.e., find optimal parameters for) an SVM model. We preprocessed the records to remove commonly used words (e.g., “and,” “the”) that appear on the PubMed “stopword” list [8]. During our initial development phase, we found that an ensemble of two SVM models with minor differences resulted in greatest accuracy when evaluated on the training data. We therefore selected an ensemble of two SVM variants for use in the study (see Fig. 2). The first classifier (SVM1) represented the texts as individual words, pairs of words, and triplets of words (*uni-, bi-, and tri-grams*). This accounts for situations in which adjacent words affect document class (e.g., the text “randomized controlled trial” might be more strongly indicative of an RCT than any word individually). The second classifier (SVM2) used a *unigram* model (i.e., each word is considered individually) and used a strategy of *oversampling*. This strategy aims to reduce the likelihood of missing a “rare class” (here the “rare class” is RCTs, which account for ~5% of the data set) by artificially increasing the number of RCTs in the training data set by random sampling with replacement (this process is not repeated with the calibration or validation data sets, which are left in their original state).

The source code for building SVM1 is available at https://github.com/alan-turing-institute/DSSG19-Cochrane/blob/dev/analyses/partner_baseline/create_model.py.

The source code for building SVM2 is available at https://github.com/ijmarshall/robotsearch.

### 2.8 Generating calibrated probability estimates

SVMs estimate the distance between a given record and a “hyperplane” [17], which, in the current use, separates RCTs (the positive class) from non-RCTs (the negative class). The hyperplane distance metric is not readily interpretable (in our data set, this metric had a numeric value approximately between −1 and +8), and we therefore sought to add probability estimates to meet the needs of Cochrane users, who have found this feature to be particularly useful in understanding the output. To achieve this, we calibrated the ensemble SVM scores on the Clinical Hedges data set using a logistic regression model (known as Platt scaling) [18]. This generated a score for each “unseen” record in the calibration or validation data sets that is bounded by 0 and 1. These scores are closer to representing the true probability that a given record reports an RCT; as such they are readily interpretable, with higher scores representing a higher likelihood that the record reports an RCT (and vice versa). When viewed graphically, the distribution of scores is often U-shaped, with most records being assigned either a high (close to 1) or low (close to 0) probability score, and a smaller number of records in the middle that are more ambiguous in terms of class membership.

### 2.9 Evaluation metrics

In this article, we use the conventional information retrieval terminology *recall* and *precision*, which are synonymous with sensitivity and positive predictive value, respectively. As outlined previously, the recall statistic is of primary concern in the current use scenario—that is, that eligible study reports are not incorrectly discarded from the Evidence Pipeline workflow. Cochrane required the system to have at least 99% recall. Recall is calculated as the proportion of relevant items (i.e., records describing an RCT) that are correctly identified by the Evidence Pipeline workflow compared with the total number of records genuinely reporting an RCT that should have been identified.

To evaluate the discriminative performance and quality of calibration of our machine learning strategy on the Clinical Hedges data, we used bootstrap sampling as described by Steyerberg et al. [19]. In short, a series of artificial new data sets were “bootstrapped” by random sampling with replacement from the Clinical Hedges dataset. Logistic regression models (which served the dual purposes of ensembling the individual SVM models and producing calibrated probability outputs) were trained on each sampled data set and evaluated on the original data set. This process was repeated 5,000 times and used to estimate performance metrics with 95% confidence intervals. Although the primary use of the system is for binary classification, a key secondary use is providing indicative probability scores to users. We evaluate the quality of the probabilities via a calibration plot and by calculation of the Brier score and C statistics [20].

Common practice in Cochrane Reviews is to find, use, and cite all published (and unpublished) reports of each included study (“study reports”). Many studies included in Cochrane Reviews comprised multiple study reports. This means that if the classifier “misses” one of several study reports of the same RCT, this does not necessarily mean the RCT study has been “lost.” We therefore adopted the following approach. We first classified all study reports in Cochrane Reviews of RCTs using the machine learning classifier and then we considered a study to be “lost” only if *all* reports of that study fell below the threshold. As such, the “study” is our unit of analysis rather than the “study report.” We made this decision because we found many secondary citations in reviews referred to indirectly related non-RCT studies and also because we would expect the retrieval of a single article would alert the review team to the existence of the trial.

Precision is also a metric of interest because it can be used to compute the number of articles requiring manual screening by Cochrane Crowd in the Evidence Pipeline workflow. Here, we were concerned with the number of irrelevant records (i.e., records not reporting an RCT) that are incorrectly classified by machine learning as relevant (i.e., records with an assigned probability score above the identified threshold score), which must then be filtered out manually by the Cochrane Crowd. Precision is calculated as the proportion of retrieved records, which genuinely report an RCT.

Recall and precision were calculated from a 2 × 2 table representing positive/negative (relevant/irrelevant) classes and whether they were correctly or incorrectly classified (Table 1).

**Table 1:** 2 × 2 table from which precision and recall are calculated

	RCTs (gold standard)	Non-RCTs (gold standard)
Machine learning classed RCTs	True positives	False positives
Machine learning classed non-RCTs	False negatives	True negatives

*Abbreviation*: RCT, randomized controlled trial.

Formulas used to calculate precision and recall are as follows:

$$\begin{array}{l} precision=\;\frac{true\;positives}{true\;positives+false\;positives}\\ recall=\;\frac{true\;positives}{true\;positives+false\;negatives} \end{array}$$

We computed statistics for precision at 99% recall against the Clinical Hedges calibration data set. As specified previously, recall was set at 99% by the IRMG. As the Cochrane Reviews we examined contain only RCTs (and the non-RCTs excluded during searches are not usually comprehensively recorded), we were not able to calculate precision on the Cochrane Reviews dataset and report recall only.

For the primary analysis, the denominator was all articles in Cochrane Reviews meeting the minimum character length criteria described previously (i.e., very short titles and abstracts were excluded). We assume that manual assessment will yield 100% recall of these records. We also report results on the full data set, without removing articles with small or nonexistent abstracts, as a secondary analysis. The first figure can be interpreted as the recall of the overall workflow because it takes account of our decision to remove records with insufficient information for machine classification from the workflow.

We present absolute values of the total number of eligible studies “lost” to Cochrane Reviews. Finally, we also present the distribution of “lost” study reports according to the year of publication because we hypothesize that the classifier may perform less well on older study reports because (1) it has been trained on newer reports and (2) trial reporting may have improved as a result of the CONSORT statement.

## 3 Results

The machine learning classifier for identifying reports of randomized trials (Cochrane RCT Classifier) was built as per the previously mentioned methods from the screening of 280,620 Embase records (January 2014 to July 2016) by Cochrane Crowd. Of these, 20,454 (7.3%) were deemed to be RCTs.

### 3.1 Threshold setting and binary classification performance

The 49,025 records from the Clinical Hedges data set were scored by the machine learning classifier. The records were ordered according to classifier score, and precision and recall statistics were calculated for every record in sequence. The classifier probability, which corresponded with 99% recall, was recorded and used as the classification threshold for the later validation (and the deployed system). The discriminative and calibrative performance of this strategy, estimated using bootstrap sampling is presented in Table 2. We estimate that precision was 8.3%, meaning that one in every 12 records retrieved described an RCT. Setting the classifier at this level of recall resulted in 58% of records in this data set being automatically discarded as highly unlikely to be reporting a randomized trial.

**Table 2:** Bootstrap estimates of model performance on Clinical Hedges data set, with 95% confidence intervals

Validation precision	Validation recall	Validation specificity	C statistic	Brier score
0.08 (0.06, 0.12)	0.99 (0.98, 0.99)	0.63 (0.48, 0.76)	0.98 (0.98, 0.98)	0.05 (0.05, 0.05)

**Fig. 3:**
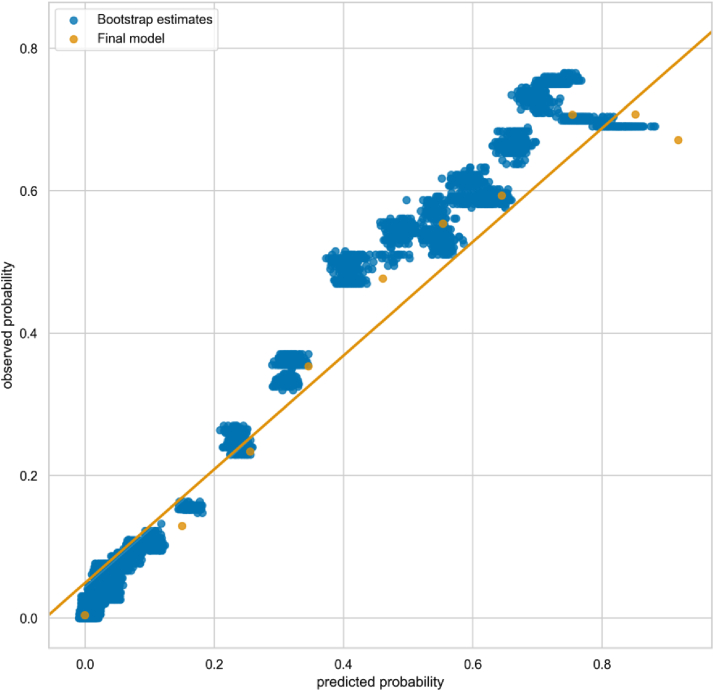
Calibration plot showing bootstrap estimates of predicted vs. observed probabilities of an article being an RCT in Clinical Hedges dataset (each blue point represents an estimate of a model generated from one bootstrap sample) and the performance of the final model (orange). (For interpretation of the references to color in this figure legend, the reader is referred to the Web version of this article.)

**Fig. 4:**
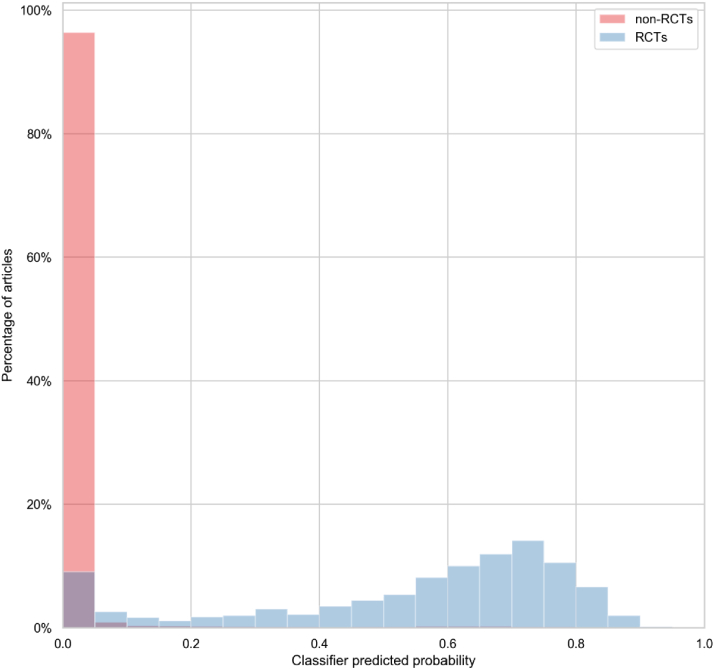
Distribution of classification scores for RCTs and non-RCTs in Clinical Hedges data set. RCT, randomized controlled trials.

Estimates of the C statistic and Brier score were 0.978 and 0.048, respectively, indicating excellent discriminative performance. We present a calibration plot showing point estimates from the bootstrap evaluation and the final model (trained on the whole dataset) in Fig. 3. We show how the predicted scores are distributed for RCTs and non-RCTs in Fig. 4.

### 3.2 Validating the classifier recall on studies included in Cochrane Reviews

The title and abstract records of 58,283 studies included in 4,296 Cochrane Reviews were fed through the classifier. Records with a score equal to or above the threshold identified in the previous step were automatically classified as potentially reporting an RCT; those scoring below this threshold were automatically classified as not reporting an RCT.

Table 3 summarizes the number of eligible studies that are “lost” to reviews as a result of all of their corresponding study reports scoring lower than the threshold. When records that contain insufficient information for machine classification are excluded from machine classification and assumed to be manually assessed (see Section 2), the classifier correctly identifies 99.5% (43,783 out of 44,007) of studies.

**Table 3:** Number of included studies in Cochrane Reviews classified as RCTs

	RCTS correctly identified by the classifier (recall)	RCTS not identified by the classifier
All studies (*N* = 58,283)	54,683 (93.8%)	3,600 (6.2%)
Studies with sufficient information for machine classification (*N* = 44,007 studies)	43,783 (99.5%)	224 (0.5%)

*Abbreviation*: RCT: randomized controlled trial.

In our secondary analysis, when we include for machine classification data for all studies (including the subset of studies, which contain insufficient information for accurate machine classification [see Section 2]), we find that 3,396 studies would be potentially “lost” to reviews (compared with 224 studies when only those with sufficient information are included).

**Fig. 5:**
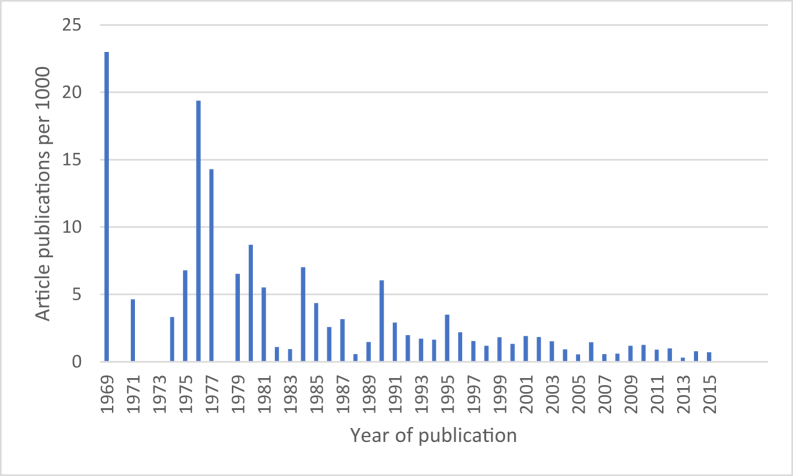
RCTs “lost” by the classifier per 1,000 published, by year of publication, showing that the risk of “losing” a publication decreases over time.

Fig. 5 shows the 224 randomized trials “lost” by the classifier per 1,000 published, by year of publication, for all but one of the publications (the age of one publication could not be ascertained). These results show that older reports are much more likely to be misclassified by the machine learning classifier.

## 4 Discussion

### 4.1 Summary of findings

We conducted a three-stage study that involved training, calibrating, and evaluating a machine learning classifier designed to distinguish between bibliographic title–abstract records that report an RCT and those that do not. Recall falls to an unacceptably low level (94%) if records with limited information in their titles and/or abstracts are submitted for machine classification. However, when these records are excluded, the classifier exceeds the standard required by Cochrane with recall at 99.5% of all those records scored. It should be noted that this means that some records are unsuitable for machine learning and so must necessarily be checked manually; however, this mirrors current practice, whereby records with limited information in their titles and abstracts are retained for further assessment on the basis of their corresponding full-text reports.

We deem the recall level as “acceptable” for use in “live” reviews on the basis that (1) this exceeds the recall of validated RCT search filters that have been used in systematic review production for many years and (b) this threshold was agreed by methodologists in Cochrane for use in Cochrane Reviews.

Although the precision of 8% estimated against the Clinical Hedges dataset appears low, this is partly because of the age of that data set and relatively low prevalence of RCTs. In the Cochrane Evidence Pipeline workflow (Fig. 1), the classifier saved Cochrane Crowd from needing to check 185,039 records manually (out of a total of 449,480) during the 2018 calendar year, a very large saving in manual workload [13].

Systematic reviews are frequently used to support decision-making processes for both policymakers and practitioners and are also key sources of evidence in drug licensing regulation. Reviews need to be accurate representations of the state of current knowledge, as decisions that are based on their findings can affect people's lives. Reviews also need to be demonstrably correct, as the way in which evidence is synthesized can have implications, for example, for drug licensing, and can therefore be open to legal challenge. These joint imperatives—for systematic reviews to be correct and to be seen to be correct—generate the normative expectation that they should contain all relevant research evidence and the corollary concern that review findings based on bodies of evidence that inadvertently exclude some eligible studies are potentially unreliable. To this end, our study provides data demonstrating the reliability of implementing what could be seen as a major innovation in study identification methods for systematic reviews, the automatic eligibility assessment of study reports, and the exclusion of a portion without any manual checking by humans, rolled out at scale across Cochrane: the largest producer of systematic reviews globally and an organization committed to minimizing the risk of bias in review production through methodological and editorial rigor. We note that the recall threshold set by Cochrane (99%) exceeds the performance of conventional search methods (e.g., the Cochrane Highly Sensitive Search Strategy was found to have recall of 98.5% by the Clinical Hedges team) [21], and we have demonstrated in previous work that our machine learning approach can exceed the precision achieved by conventional search filters [8].

Although our results indicated that 0.5% of studies could have been “lost” to Cochrane Reviews if authors had used this classifier (affecting 178 reviews, leaving 4,118 reviews unaffected), this is almost certainly an overestimate when considering the prospective use of this classifier to support the identification of newly published RCTs for new, updated, and/or living systematic reviews. First, other means of finding studies are routinely used in Cochrane Reviews alongside conventional electronic searching—such as checking reference lists and citing records or contacting researchers who are active in the topic area—so some of these “lost” studies would likely be found using these complementary search methods. Second, studies that are potentially lost are overwhelmingly older reports. Although we do not dismiss the potential importance of identifying older trials for consideration in systematic reviews, it is reassuring that more recent studies (relevant especially for newer treatments and review updates) are far less likely to be missed. One reason the classifier performs better for more recent studies could be improvement in the reporting of RCTs over time, for example, in response to the CONSORT statement [16,22]. Trialists are now widely expected to detail trial methodology in the report's abstract and to include the fact that they are reporting an RCT in its title.

### 4.2 Strengths and weaknesses of this evaluation

In this article, we have described a robust evaluation of the performance of an RCT classifier in a large data set of systematic reviews. We were fortunate in having three large, independently generated, high-quality data sets available to train, calibrate, and validate the classifier. This is an unusual position to be in, and there are probably few study designs other than RCTs with comparably high-quality data sets available. We note that this may limit the potential to evaluate the performance of similar workflows, created to identify other types of study design, using the same three-stage process.

The current classifier has been trained almost exclusively on records published in English, so it does not necessarily generalize to other languages. However, this important limitation is, in principle, surmountable, as machine learning technology is language agnostic and would therefore be capable of modeling any language, so long as sufficient training data were available.

The focus of this work has been to build a machine learning classifier for deployment in a specific workflow. The machine learning classifier we have developed meets required levels of recall but inevitably results in some studies being “lost” to reviews. This study does not attempt to ascertain the impact of these losses on the affected reviews' statistical and narrative results and findings, and a future extension of this study will investigate this important question. We also note that only 178 of 4,296 reviews were affected, leaving results unchanged in at least 96% of the reviews.

### 4.3 Next steps: the “Screen4Me” service

We are currently piloting an extension to the Evidence Pipeline for use with individual Cochrane Reviews. Authors using this service will compile their set of potentially eligible records from searches of multiple databases (including CENTRAL) as is typical for any systematic review. Given that the RCT classifier and Cochrane Crowd have already classified more than 800,000 study records (and increasing by >10,000 per month), it is likely that a proportion of the records retrieved and uploaded to the Classifier by authors have already been classified according to whether they report an RCT or not. Where this is the case, the records that are already known not to describe RCTs will be removed from the workflow. The remaining studies will then be sent to the RCT classifier, and those records classified as not reporting an RCT (i.e., that fall below the 99% recall threshold) will be discarded. Finally, the records classified as potentially reporting an RCT will be screened by Cochrane Crowd. The review team is then left with a much smaller pool of records to examine, containing only RCTs. In early pilots, this new workflow reduced manual screening workload by between 40% and 70% depending on the prevalence of RCTs in the search results of individual reviews.

## 5 Conclusions

The Cochrane RCT Classifier is now deployed by Cochrane for reducing screening workload in review production. As part of a wider workflow that includes prospective database searches and crowdsourcing to build a comprehensive database of RCTs, machine learning can reduce the manual screening burden associated with research synthesis while ensuring a very high level of recall that is acceptable for an organization, which depends on having comprehensive access to the published research that falls within health care topics relevant to its scope.

## References

[1] Cochrane (2019). Cochrane Library. https://www.cochranelibrary.com/.

[2] Lefebvre C., Glanville J., Briscoe S., Littlewood A., Marshall C., Metzendorf M., Higgins J., Thomas J., Chandler J., Cumpston M., Li T., Page M. (2019). Chapter 4: searching for and selecting studies. Cochrane Handbook for Systematic Reviews of Interventions.

[3] Bastian H., Glasziou P., Chalmers I. (2010). Seventy-five trials and eleven systematic reviews a day: how will we ever keep up?. PLoS Med.

[4] Shojania K.G., Sampson M., Ansari M.T., Ji J., Doucette S. (2007). How quickly do systematic reviews go out of date? A survival analysis. Ann Intern Med.

[5] Macleod M.R., Michie S., Roberts I., Dirnagl U., Chalmers I., Ioannidis J.P.A. (2014). Biomedical research: increasing value, reducing waste. Lancet.

[6] Tsafnat G., Dunn A., Glasziou P., Coiera E. (2013). The automation of systematic reviews. BMJ.

[7] O’Mara-Eves A., Thomas J., McNaught J., Miwa M., Ananiadou S. (2015). Using text mining for study identification in systematic reviews: a systematic review of current approaches. Syst Rev.

[8] Marshall I., Storr A.N., Kuiper J., Thomas J., Wallace B.C. (2018). Machine learning for identifying randomized controlled trials: an evaluation and practitioner’s guide. Res Synth Methods.

[9] Wallace B.C., Noel-Storr A., Marshall I.J., Cohen A.M., Smalheiser N.R., Thomas J. (2017). Identifying reports of randomized controlled trials (RCTs) via a hybrid machine learning and crowdsourcing approach. J Am Med Inform Assoc.

[10] Thomas J., Noel-Storr A., Marshall I., Wallace B., McDonald S., Mavergames C. (2017). Living systematic reviews: 2. Combining human and machine effort. J Clin Epidemiol.

[11] Cochrane (2019). About the CRS (Cochrane Register of Studies). Cochrane Community. https://community.cochrane.org/help/tools-and-software/crs-cochrane-register-studies/about-crs.

[12] Noel-Storr A., Dooley G., Wisneiwski S., Glanville J., Thomas J., Cox S. (2020). Cochrane Centralised Search Service showed high sensitivity identifying randomized controlled trials: a retrospective analysis. J Clin Epidemiol.

[13] Noel-Storr A, Dooley G, Elliott J, Steele E, Shemilt I, Mavergames C, et al. An evaluation of Cochrane Crowd finds that crowdsourcing can help to address the challenge of information overload in evidence synthesis. *J Clin Epidemiol*. 10.1016/j.jclinepi.2021.01.006 33476769

[14] Nevin L. (2018). Advancing the beneficial use of machine learning in health care and medicine: toward a community understanding. PLoS Med.

[15] Wilczynski N.L., Douglas M., Haynes R.B., Team and the H (2005). An overview of the design and methods for retrieving high-quality studies for clinical care. BMC Med Inform Decis Mak.

[16] Schulz K.F., Altman D.C., Moher D. (2010). CONSORT 2010 Statement: updated guidelines for reporting parallel group randomised trials. BMC Med.

[17] Sain S.R., Vapnik V.N. (2006). The nature of statistical learning theory.

[18] Platt J.C. (1999). Probabilistic outputs for support vector machines and comparisons to regularized likelihood methods. Adv Large Margin Classif.

[19] Steyerberg E.W., Harrell F.E., Borsboom G.J.J.M., Eijkemans M.J.C., Vergouwe Y., Habbema J.D.F. (2001). Internal validation of predictive models: efficiency of some procedures for logistic regression analysis. J Clin Epidemiol.

[20] Brier G. (1950). Verification of forecasts expressed in terms of probability. Mon Weather Rev.

[21] McKibbon K.A., Lou W.N., Haynes R.B. (2009). Retrieving randomized controlled trials from MEDLINE: a comparison of 38 published search filters. Health Info Libr J.

[22] Turner L., Shamseer L., Altman D.G., Schulz K.F., Moher D. (2012). Does use of the CONSORT statement impact the completeness of reporting of randomised controlled trials published in medical journals? A Cochrane review. Syst Rev.

